# Response: Commentary: The Impact of the Time Interval Between Radiation and Hyperthermia on Clinical Outcome in Patients With Locally Advanced Cervical Cancer

**DOI:** 10.3389/fonc.2020.00528

**Published:** 2020-04-15

**Authors:** Johannes Crezee, Arlene L. Oei, Nicolaas A. P. Franken, Lukas J. A. Stalpers, H. Petra Kok

**Affiliations:** ^1^Department of Radiation Oncology, Amsterdam University Medical Centers, University of Amsterdam, Amsterdam, Netherlands; ^2^Laboratory of Experimental Oncology and Radiobiology, Amsterdam University Medical Centers, University of Amsterdam, Amsterdam, Netherlands; ^3^Center for Experimental Molecular Medicine, Amsterdam University Medical Centers, University of Amsterdam, Amsterdam, Netherlands

**Keywords:** radiotherapy, hyperthermia, time interval, DNA damage repair, reoxygenation

Mild hyperthermia (39–43°C) is an effective radiosensitizer (1). Hyperthermia can eradicate tumor cells, particularly hypoxic cells, independently from timing with radiotherapy, with a clear dose-effect relationship (2–4). Hyperthermia also synergizes more directly with radiotherapy. Temperatures exceeding 39°C cause induction of heat shock proteins (HSPs), formation of reactive oxygen species (ROS), decrease in Superoxide dismutase (SOD), enhanced tissue perfusion and reduced oxygen consumption, the latter leading to reoxygenation and enhanced effectiveness of radiotherapy for hyperthermia given before radiotherapy (5–11). Temperatures exceeding 41°C cause inhibition of multiple repair pathways of radiotherapy-induced DNA damage if hyperthermia is given shortly before or after radiotherapy (1, 12, 13). Aforementioned effects all require different timing, sequence and temperature levels, and both time-dependent synergistic and time-independent additive hyperthermic effects contribute to the effectiveness of clinical hyperthermia (14) as confirmed in an *in vivo* mouse tumor model (15).

Hyperthermia is clinically proven radiosensitizer for many tumor sites (16, 17), including locally advanced cervical cancer (LACC) (18, 19). Recently two groups analyzed the impact of time interval on clinical outcome for LACC patients similarly treated with radiotherapy, followed by hyperthermia once a week. Long time intervals are possibly less effective, as radiotherapy-induced DNA damage will get repaired within hours after radiotherapy and for long intervals hyperthermia-induced inhibition of DNA repair can simply come too late to have a therapeutic effect. Our group did find such an impact (20), whereas Kroesen et al. did not (21), sparking a debate on potential reasons including tumor temperature (22, 23).

We have therefore read with interest the response of Kroesen et al. (22) on our opinion article “The Impact of the Time Interval between Radiation and Hyperthermia on Clinical Outcome in Patients with Locally Advanced Cervical Cancer” (23), and appreciate their thorough efforts to analyze possible effects of the time interval between radiotherapy and hyperthermia on treatment outcome in LACC patients treated with radiotherapy followed by hyperthermia. They now specifically checked this impact in a subgroup of their cohort in which the highest temperature was measured. They did so as van Leeuwen et al. had reported slightly higher tumor temperatures and because their group had reported preclinical results showing a strong dose-effect relationship in inducing inhibition of homologous recombination DNA repair, yielding stronger radiosensitization at higher temperatures (12). However, they could not find any indication that shorter time intervals were clinically more effective than longer intervals, not even in the subgroup with the highest temperatures.

Though looking similar, there may still be differences between the two studies preventing a direct clinically relevant comparison. Among these the fact that higher tumor temperatures were achieved in our group (23), and also the definition of the time interval was different in both studies. Kroesen et al. defined the time interval between EBRT and HT as the time between the first beam-on of the radiotherapy treatment and the start of the heating, that is switching power-on on the HT device (21). This means that fully therapeutic temperature levels exceeding 41°C are probably reached 15–30 min later. In our cohort the time interval was defined as the time between the end of RT and the moment during HT that 41°C was reached, thus our reported time intervals include the initial 15–30 min warming-up period during the preparation phase of hyperthermia treatment. This was chosen as no inhibition of DNA damage repair will occur when tumor temperatures have not yet reached 41°C (20). Our reported time intervals are thus effectively 15–30 min shorter than the intervals reported by Kroesen et al. (21): their “short” interval is close to our “long” time interval. This difference may be clinically relevant depending on how fast DNA repair takes place in the clinical situation. Van Leeuwen et al. established for tumor biopsies of LACC patients, taken at different times after radiotherapy, that all DNA damage is repaired within 2 h after radiotherapy (20), meaning hyperthermia must be given within 1 h. In contrast, DNA damage had only partially been repaired 4 h after radiotherapy in *in vitro* studies in three cervical cancer cell lines (24).

There might be a rationale to consider reversing the sequence and first give hyperthermia, followed by radiotherapy in LACC patients. Reversing sequence yields considerably shorter time intervals become technically feasible as it is no longer needed to heat up the tumor first, which takes at least 15–30 min for deep-seated tumors. Thus, more hyperthermia effects can contribute, including reoxygenation already effective at temperatures below 41°C (5–7). For reversed order the duration of BRCA2 depletion determines the effectiveness of that particular hyperthermia effect, independent of the rate of DNA repair after radiotherapy. This depletion may last longer than the rate with which DNA damage gets repaired. Hypofractionation would increase the number of hyperthermia fractions combined with radiotherapy, while in both LACC protocols hyperthermia was added only once a week. Clinical feasibility of applying such a reversed sequence with hypofractionation and much shorter time intervals has been demonstrated for treatment of recurrent breast cancer (25). Caution is needed when considering optimal sequence and timing for other tumors and combination with radiochemotherapy.

While awaiting more data on clinical DNA repair rates we can conclude that both studies present valid clinical results. Thus, longer time intervals between radiotherapy and hyperthermia are not detrimental for treatment outcome and referral of LACC patients from centers without hyperthermia facilities for hyperthermia treatment in other centers is acceptable. The possible explanation: hyperthermia exhibits multiple working mechanisms, including -besides inhibition of DNA repair- tumor reoxygenation and direct cytotoxicity. Many mechanisms remain fully effective with longer time intervals between radiotherapy and hyperthermia (5–7, 13–15). This is also clinically evident for LACC patients, as also with a long time interval the effective contribution of hyperthermia to radiotherapy is of similar magnitude as the contribution of adding chemotherapy to radiotherapy, as demonstrated in the RADCHOC study (26).

So referral for hyperthermia of LACC patients who continue to receive radiotherapy at referring centers is clinically acceptable. We also subscribe to the remark of Kroesen et al. that the strong dose-effect relationship found for hyperthermia in several clinical studies is proof that hyperthermia yields a clinically relevant benefit (20, 27, 28).

## Author Contributions

JC and HK contributed conception and design of the study. JC wrote the first draft of the manuscript. HK wrote sections of the manuscript. All authors contributed to manuscript revision, read, and approved the submitted version.

### Conflict of Interest

The authors declare that the research was conducted in the absence of any commercial or financial relationships that could be construed as a potential conflict of interest. The reviewer MH declared a past co-authorship with several of the authors JC and HK to the handling Editor.
